# Enhancing fruit quality attributes and storability of Ruby Seedless grape cultivar using different irradiated potassium sources as an alternative to ethrel

**DOI:** 10.1186/s12896-025-01082-1

**Published:** 2025-12-17

**Authors:** Mohamed Farouk Ahmed, Ola Mohamed Fekry, Basma Salah Eldin Ahmed Salama

**Affiliations:** 1 Natural Products Department, National Centre for Radiation Research and Technology, Egyptian Atomic Energy Authority, Cairo, Egypt; 2 Fruit Handling Research Department, Horticulture Research Institute, Agricultural Research Center, Giza, Egypt; 3 Horticulture Department, Faculty of Agriculture, Menofia University, Shebin El-Kom, Menofia, Egypt

**Keywords:** Grape, Ionizing radiation, Foliar, Weight loss, Anthocyanin, Shattering

## Abstract

The current study aims to assess the efficiency of different irradiated potassium sources as alternatives to ethrel in enhancing the fruit quality and storability of Ruby grapevines. This investigation was conducted on seven- year- old Ruby Seedless grape cultivars that were sprayed as follows: control (water); ethrel at 150 ppm; potassium citrate at 2000 and 4000 ppm; irradiated potassium citrate at 750 and 1500 ppm; potassium silicate at 2000 and 4000 ppm and irradiated potassium silicate at 750 and 1500 ppm. The data revealed that all the potassium sprays used significantly increased cluster weight, berry weight, and berry firmness compared with those in the control and ethrel groups. Moreover, the total soluble solids, sugars and anthocyanin contents increased with decreasing acidity% compared with those of the control group. Storability data revealed that all potassium sprays decreased weight loss, decay, firmness loss, and shattering during the storage period compared with those of the control and ethrel groups. Additionally, the total soluble solids, sugars and anthocyanin contents increased with decreasing acidity compared with those of the control. In conclusion, potassium sprays, especially irradiated potassium silicate, can be a good alternative to ethrel for improving the physical and chemical properties of Ruby Seedless grapes and increasing their storability by improving firmness and reducing weight loss, decay, and shattering.

## Introduction

Ruby Seedless is a late-season table grape cultivar with oval berries, a reddish-purple color, and high bud fertility. However, the primary issue with its production, is that it does not reach the required degree of redness, most likely because of the over cropping phenomenon, summertime high temperatures, and the limited day/night temperature range, which prevents anthocyanin accumulation [1]. Clusters of inconsistent hues are signs of low-quality grapes, which cause producers to harvest more than once, increasing production expenses. The use of growth regulators such as ethylene during berry ripening is one of the methods used to address or avoid these issues [2]. Exogenously applied ethylene promotes the expression of genes related to the formation of anthocyanin, which improves berry skin color and accelerates grape maturation [3]. Ethrel, a plant growth regulator that releases ethylene, functions similarly to the phytohormones that vines naturally make. It accelerates fruit ripening, changes plant metabolism, and enhances grape color [4]. On the other hand, ethrel applied too late or in excess may result in soft berries with a limited shelf life [5]. Furthermore, ethrel may have a toxic effect on human health [6]. Therefore, it is increasingly important to explore new methods for improving fruit color while reducing or eliminating the use of synthetic chemicals that support sustainable fruit production methods. Consequently, it is imperative to explore natural alternatives to alleviate the risks associated with using ethrel to enhance fruit color.

One of the most effective and commonly used approaches to enhance nutrient uptake and improve tree health is foliar nutrient supplementation. Fruit trees have demonstrated the efficiency of foliar treatment because of their deep roots, the fact that most fertilizers applied to the soil surface are depleted, and their inability to reach the root area effectively nourishes plants [7]. Potassium (K) is one of the most crucial fertilizer components that influence the quality of fruit. It has also been shown to be involved in osmoregulation, cell expansion, stomatal movement, and photosynthesis [8]. Additionally, it is the most abundant cation and only exists as a free ion. It activates several enzymes, some of which are responsible for solute transport, energy metabolism, and protein synthesis [9]. According to Adrees et al. [10], silicon (Si) is one of the most essential key elements needed for plant growth because it is required for numerous physiological processes. The most significant of which are improving the efficiency of photosynthesis, the effectiveness of antioxidant enzymes, and the efficiency with which roots absorb nutrients needed for plant growth and development. Anecdotal data indicate that the use of silicon sprays may “strengthen” the plant by thickening its cell walls and improving its ability to absorb light. This results in the growth of fruit and seeds with greater flavor, aroma, color, and nutritional value [11, 12]. Citric acid is an essential natural antioxidant and stress-reduction tool that also functions remarkably well as a molecular messenger in certain defensive mechanisms and physiological activities of plants [13]. According to recent studies, the use of citric acid enhances the quality of table grapes, reduces weight loss, preserves fruit firmness, maintains the integrity of the membrane, and prolongs fruit storage life [14].

Irradiation produces ionizing free radicals in materials, increasing their effectiveness for their intended purpose [15]. The use of radiation, particularly gamma rays, is an efficient and rapid method for ionizing complex compounds and increasing their availability [16, 17]. Compared with those of non-irradiated potassium compounds, the release rates of irradiated potassium compounds were greater [18]. Thus, the present study aims to evaluate the efficiency of different irradiated potassium sources as alternatives to ethrel in enhancing the fruit quality and storability of Ruby Seedless grapevines.

## Material and methods

### Plant material and treatments

This investigation was conducted for two consecutive seasons 2022 and 2023 in a private vineyard located on the Cairo–Alexandria desert road, 58 km from Cairo, Egypt (30°14′12.6″N, 30°51′33.1″E), to study the feasibility of using different levels of various irradiated potassium sources as alternatives to ethrel on seven-year-old Ruby Seedless grape cultivars. The owner of the farm provided us with permission to perform the study, which we did in compliance with local laws. Vines were grown 2.5*3 m apart in sandy soil with the drip system and trained with the bilateral cordoning system. The vines were pruned to spurs and trellised according to the telephone system during the third week of January with a bud load of 24 buds/vine, resulting in an average of 30–32 clusters/vine.

The vines were all of similar vigor and received standard horticultural techniques already in use in vineyards according to the Egyptian Ministry of Agriculture. In addition to the standard horticultural practices, the other vines, aside from the control, were treated with ethrel at 150 ppm; potassium citrate at 2000 ppm (KC1) and 4000 ppm (KC2); irradiated potassium citrate at 750 ppm (γKC1) and 1500 ppm (γKC2); potassium silicate at 2000 ppm (KSi1) and 4000 ppm (KSi2); and irradiated potassium silicate at 750 ppm (γKSi1) and 1500 ppm (γKSi2). The 2000 ppm dose was chosen according to Okba et al. [19], and we used the 4000 ppm dose to study whether there is scope for using higher doses with better results. For irradiated sprays, we reduced the dose according to Trangia & Macusi [20] to avoid the possibility of toxicity due to limited data on irradiated potassium. The study included one hundred and twenty uniform vines. Every four vines were used as a replicate and each treatment consisted of three replicates. All the treatments were foliar sprayed twice: after the fruit set stage, when the berry diameter reached 2–3 mm and at the veraison stage (20% berry color), the tested trees were sprayed to the drip point, whereas in the ethrel treatment, the clusters were sprayed once at the veraison stage (20% berry color). All the conducted treatments, as well as the control had Tween-80 (0.5% v/v) to enhance the wetting qualities of the solutions and their adhesion to the surface of the leaves or fruits.

### Chemicals

Potassium citrate and potassium silicate grade standards (purity > 95%) and ethrel (48% ethephon) were supplied by Sigma‒Aldrich (St. Louis, MO, USA) along with other chemicals and reagents utilized in the study.

### Irradiation process

Potassium citrate and potassium silicate aqueous solutions (1 mol.) were irradiated at a dose of 10 kilo gray (kGy) via a Gamma Cell (60Co) at the National Center for Radiation Research and Technology, Egyptian Atomic Energy Authority, Cairo, Egypt (dose rates of 0.76 and 0.67 kGy/h for 2022 and 2023, respectively).

### Parameters

- At- harvest parameters

Representative random samples of twelve clusters/vine were harvested at maturity when the total soluble solids (TSS) content reached 16–17% and transferred to the laboratory to determine fruit quality attributes [21].

1- Physical parameters: cluster weight (g), berry weight (g), and firmness (g/cm^2^).

2- Chemical parameters: TSS (%), total acidity (g tartaric acid/100 ml juice) and total sugars (%) were determined as described by the AOAC [22]. Additionally, total anthocyanin (mg/100 g FW) was identified as described by Fuleki and Francis [23].

- Storability parameters

Clusters of each treatment were harvested and placed in three carton boxes (the 1^st^ for the loss of weight, the 2^nd^ for decay and the last for fruit quality traits at different sampling dates: 0, 1, 2, 3, and 4 weeks of cold storage); each box was replicated three times, and each replicate consisted of four clusters. All the treatments were cold stored at 0 ± 1 °C and 90–95% relative humidity. The measured fruit physical and chemical properties during the period of storage were illustrated in Table 1.

**Table 1:** The measured fruit physical and chemical properties of ruby seedless grapevines over a four-week storage period

Fruit physical properties	Fruit chemical properties
• Weight loss% = weight loss (g)/initial cluster weight (g) × 100.	• TSS (%) on each sampling date.
• Decay percentage = weight of the decayed berries/initial cluster weight (g) × 100.	• Total acidity (%) on each sampling date
• Shattering percentage = weight of the shattered berries/initial cluster weight (g) × 100.	• Total sugars (%) on each sampling date
• Berry firmness (g/cm^2^) on each sampling date.	• Total anthocyanin (mg/100 g FW) on each sampling date

### Trial design and statistical analysis

For this experiment, a completely randomized design was used (10 treatments × 3 replicates × 4 samples/replicate). The statistical analysis of the current data was performed via analysis of variance in accordance with Snedecor and Cochran [24]. At the 5% level, averages were compared via Duncan’s multiple range tests [25]. The statistical analysis was conducted via the M-STAT computer program in accordance with McLain [26].

## Results

**Fig. 1:**
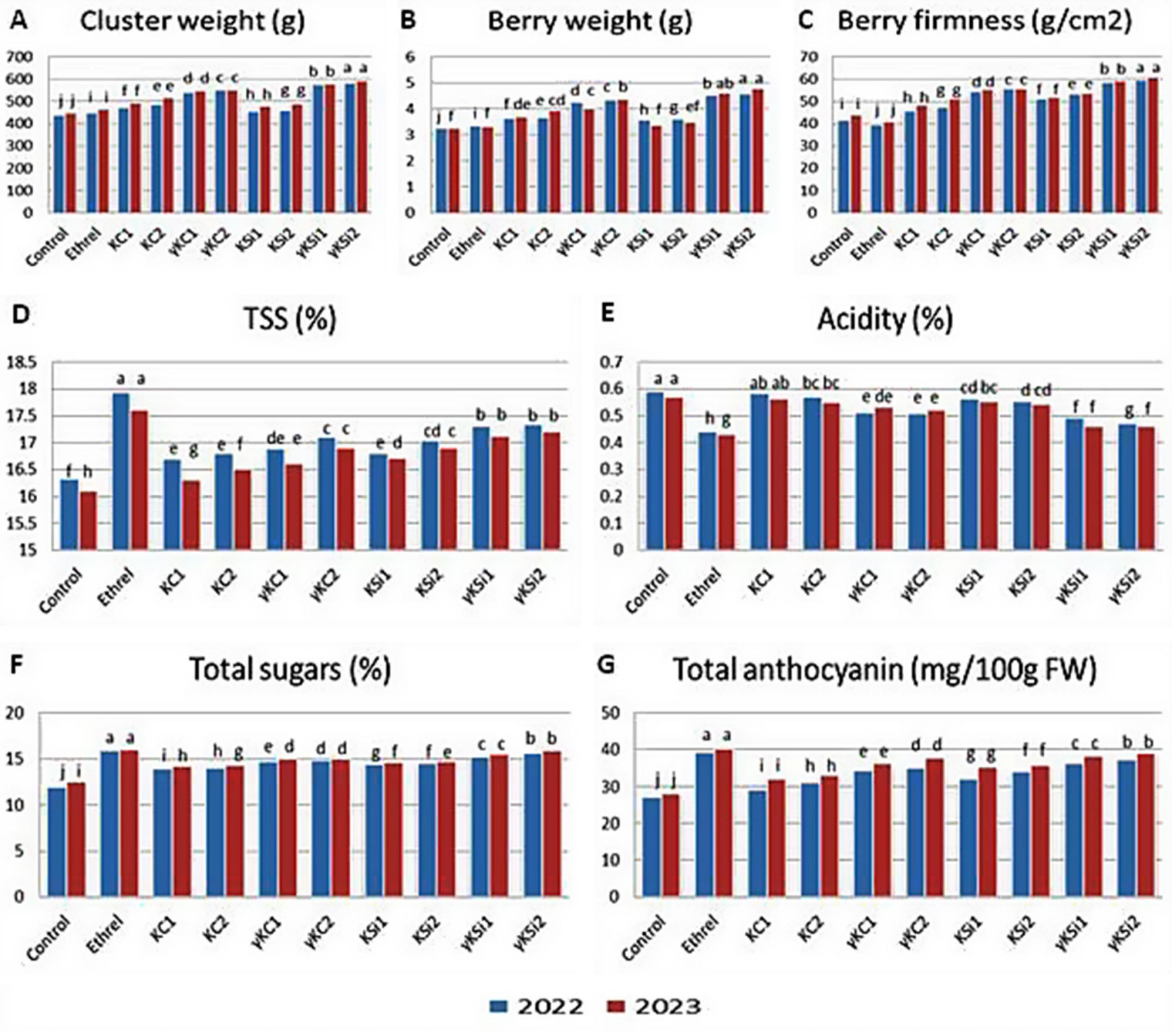
Effects of different foliar spray treatments on the cluster weight, berry weight, firmness, TSS, acidity, total sugars and total anthocyanin contents of Ruby Seedless grapevines. Means followed by different letters are significantly different at the *p* ≤ 0.05 level; Duncan’s multiple range test. KC1: potassium citrate at 2000 ppm; KC2: potassium citrate at 4000 ppm; γKC1: irradiated potassium citrate at 750 ppm; γKC2: irradiated potassium citrate at 1500 ppm; KSi1: potassium silicate at 2000 ppm; KSi2: potassium silicate at 4000 ppm; γKSi1: irradiated potassium silicate at 750 ppm; γKSi1: irradiated potassium silicate at 1500 ppm

A) Effects of ethrel and potassium sprays on cluster weight, berry weight, and berry firmness:

Figure 1 (A, B and C) shows the effects of spraying with ethrel or different potassium forms on the cluster weight, berry weight, and firmness of Ruby Seedless grapevines. The data revealed that, compared with the control, the ethrel treatment significantly increased the cluster and berry weights with exception of berry weight, in 2023. Compared with control and ethrel treatments, the different potassium spray treatments significantly increased the cluster weight and berry weight, except for berry weight with non-irradiated K silicate treatment in 2023. Compared with the K silicate spray, the non-irradiated K citrate spray yielded better results, whereas the irradiated K silicate spray outperformed the irradiated K silicate spray. In terms of berry firmness, while ethrel significantly reduced berry firmness compared with the control, all potassium sprays markedly increased berry firmness compared with the control and ethrel groups. Sprays of K silicate in both forms (non-irradiated and irradiated) have a greater effect than K citrate. The best results obtained for the abovementioned parameters were irradiated K silicate, particularly at higher level followed by irradiated K citrate, especially at high levels, non-irradiated K citrate, and then non-irradiated K silicate

### Comparison between high and low concentration levels and irradiated and non-irradiated forms

A high level of either K citrate or K silicate sprays increased the cluster weight, and berry weight and firmness more than low levels. Moreover, irradiated forms of both K citrate and K silicate sprays increased the cluster weight, and berry weight and firmness more effectively than non-irradiated forms did.

B) **Effects of ethrel and potassium sprays on the TSS, acidity, total sugars and anthocyanin contents:**

The TSS, acidity, total sugars, and anthocyanin contents of Ruby Seedless grapevines were positively affected by foliar spray of ethrel and various potassium sources, according to the data in Fig. 1 (D, E, F, and G). Compared with the control, spraying with ethrel or all the different K sprays significantly increased the TSS, total sugars and anthocyanin contents while reducing acidity. However, ethrel was the most effective treatment and outperformed all the other K sprays used. Sprays of K in the form of silicate had a more positive effect on the abovementioned parameters than did the citrate forms. The best results were obtained with ethrel spray, followed by irradiated K silicate sprays and then irradiated K citrate sprays.

### Comparison between high and low concentration levels and irradiated and non-irradiated forms

The effects of the high levels of both forms of K (citrate and silicate) on the TSS, total sugars and anthocyanin contents were greater than those of the lower levels. With respect to acidity, there was no significant difference between high and low levels of both K forms. Additionally, sprays of irradiated K forms outperformed those of non-irradiated forms in terms of all the previously mentioned parameters.

**C) Effects of ethrel and potassium sprays on weight loss and decay during storage:**

The impact of foliar spray of ethrel or different potassium sources on the post-harvest weight loss percentage and decay percentage of Ruby Seedless grapevines are displayed in Table 2. In general, the weight loss percentage increased significantly over time in the all treatments, with the maximum weight loss occurring at the end of the experiment (4^th^ week). The highest average weight loss percentage was 4.11% between the 3^rd^ and 4^th^ weeks, 1.39% between the 2^nd^ and 3^rd^ weeks, 1.31% between the 1^st^ and 2^nd^ weeks and 0.56% within the first week of storage. Among the foliar sprays used, ethrel resulted in the greatest weight loss, while all K foliar sprays significantly reduced the weight loss compared with the control and ethrel groups.

**Table 2:** Effects of spraying with ethrel or different potassium sources on the post-harvest weight loss (%) and decay (%) of Ruby Seedless grapevines over a four-week storage period

	**2022**						**2023**					
	**Weight loss (%)**											
**Weeks**	**0**	**1**	**2**	**3**	**4**	**Mean**	**0**	**1**	**2**	**3**	**4**	**Mean**
**Control**	0.00^w^	0.97^s-u^	2.34^n-p^	4.10^i^	9.22^b^	**3.33**^**B**^	0.00^x^	0.91^tu^	2.26^m-o^	3.92^g^	8.82^b^	**3.18**^**B**^
**Ethrel**	0.00^w^	1.17^r-t^	3.61^ij^	6.28^ef^	14.25^a^	**5.06**^**A**^	0.00^x^	1.23^st^	3.68^gh^	6.49^d^	14.92^a^	**5.27**^**A**^
**KC1**	0.00^w^	0.66^t-v^	1.91^o-q^	3.32^jk^	7.55^c^	**2.69**^**C**^	0.00^x^	0.72^uv^	1.97^o-q^	3.47^g-i^	6.79^d^	**2.59**^**C**^
**KC2**	0.00^w^	0.61^t-w^	1.84^pq^	3.21^j-l^	7.26^cd^	**2.59**^**CD**^	0.00^x^	0.64^u-w^	1.88^o-q^	3.27^h-j^	7.36^c^	**2.63**^**C**^
**γKC1**	0.00^w^	0.37^u-w^	1.51^q-s^	2.63^l-n^	5.98^fg^	**2.10**^**FG**^	0.00^x^	0.39^v-x^	1.53^q-s^	2.67^k-m^	6.01^e^	**2.12**^**EF**^
**γKC2**	0.00^w^	0.31^vw^	1.43^q-s^	2.48^m-o^	5.63^gh^	**1.97**^**GH**^	0.00^x^	0.35^vx^	1.47^q-s^	2.55^l-n^	5.75^e^	**2.03**^**F**^
**KSi1**	0.00^w^	0.51^u-w^	1.70^qr^	2.96^k-m^	6.72^de^	**2.38**^**DE**^	0.00^x^	0.55^u-w^	1.76^o-r^	3.06^i-k^	6.87^d^	**2.45**^**CD**^
**KSi2**	0.00^w^	0.45^u-w^	1.62^qr^	2.82^k-n^	6.39^ef^	**2.26**^**EF**^	0.00^x^	0.48^u-x^	1.67^p-s^	2.87^j-l^	6.48^d^	**2.30**^**DE**^
**γKSi1**	0.00^w^	0.25^vw^	1.35^q-s^	2.34^m-p^	5.31^h^	**1.85**^**GH**^	0.00^x^	0.20^wx^	1.27^r-t^	2.22^m-o^	4.98^f^	**1.74**^**G**^
**γKSi2**	0.00^w^	0.23^vw^	1.32^q-s^	2.29^n-p^	5.20^h^	**1.81**^**H**^	0.00^x^	0.17^wx^	1.24^st^	2.15^n-p^	4.83 ^f^	**1.68**^**G**^
**Mean**	**0.00 E**	**0.55 D**	**1.86 C**	**3.24 B**	**7.35 A**		**0.00 E**	**0.56 D**	**1.87 C**	**3.27 B**	**7.28 A**	
	**Decay (%)**											
**Weeks**	**0**	**1**	**2**	**3**	**4**	**Mean**	**0**	**1**	**2**	**3**	**4**	**Mean**
**Control**	0.00^q^	0.29^l-q^	0.53^k-q^	1.48^j^	4.72^e^	**1.41**^**B**^	0.00^l^	0.34^i-l^	0.47^i-l^	2.53^h^	4.58^e^	**1.59**^**B**^
**Ethrel**	0.00^q^	11.35^d^	26.82^c^	41.27^b^	52.40^a^	**26.37**^**A**^	0.00^l^	17.41^d^	28.79^c^	43.25^b^	55.21^a^	**28.93**^**A**^
**KC1**	0.00^q^	0.14^n-q^	0.21^m-q^	1.22^jk^	3.87^f^	**1.09**^**C**^	0.00^l^	0.19^j-l^	0.32^i-l^	1.26^i^	4.04^ef^	**1.16**^**C**^
**KC2**	0.00^q^	0.09^o-q^	0.17^n-q^	1.17^jk^	3.74^fg^	**1.03**^**CD**^	0.00^l^	0.12^kl^	0.23^j-l^	1.20^ij^	3.81^e-g^	**1.07**^**CD**^
**γKC1**	0.00^q^	0.00^q^	0.05^pq^	0.96^j-m^	3.06^g-i^	**0.81**^**CD**^	0.00^l^	0.00^l^	0.09^kl^	0.98^i-l^	3.12^f-h^	**0.84**^**CD**^
**γKC2**	0.00^q^	0.00^q^	0.04^pq^	0.92^j-n^	2.89^hi^	**0.77**^**CD**^	0.00^l^	0.00^l^	0.07^kl^	0.93^i-l^	2.98^gh^	**0.80**^**CD**^
**KSi1**	0.00^q^	0.07^o-q^	0.16^n-q^	1.07^jk^	3.45^f-h^	**0.95**^**CD**^	0.00^l^	0.08^kl^	0.19^j-l^	1.12^i-k^	3.57^fg^	**0.99**^**CD**^
**KSi2**	0.00^q^	0.04^pq^	0.07^o-q^	1.03^j-l^	3.28^f-i^	**0.89**^**CD**^	0.00^l^	0.00^l^	0.09^kl^	1.05^i-l^	3.37^f-h^	**0.90**^**CD**^
**γKSi1**	0.00^q^	0.00^q^	0.03^pq^	0.86^j-o^	2.75^hi^	**0.73**^**D**^	0.00^l^	0.00^l^	0.05^l^	0.81^i-l^	2.59^h^	**0.69**^**D**^
**γKSi2**	0.00^q^	0.00^q^	0.01^q^	0.83^j-p^	2.67^i^	**0.70**^**D**^	0.00^l^	0.00^l^	0.01^l^	0.78^i-l^	2.51^h^	**0.66**^**D**^
**Mean**	**0.00**^**E**^	**1.20**^**D**^	**2.81**^**C**^	**5.08**^**B**^	**8.28**^**A**^		**0.00**^**E**^	**1.82**^**D**^	**3.03**^**C**^	**5.39**^**B**^	**8.58**^**A**^	

Different small letters indicate significant differences between interactions at *p* ≤ 0.05; different capital letters in rows and columns indicate significant differences between groups and storage periods at *p* ≤ 0.05; Duncan’s multiple range test. KC1: potassium citrate at 2000 ppm; KC2: potassium citrate at 4000 ppm; γKC1: irradiated potassium citrate at 750 ppm; γKC2: irradiated potassium citrate at 1500 ppm; KSi1: potassium silicate at 2000 ppm; KSi2: potassium silicate at 4000 ppm; γKSi1: irradiated potassium silicate at 750 ppm; γKSi1: irradiated potassium silicate at 1500 ppm.

Moreover, K silicate sprays outperformed K citrate sprays with a lower rate of weight loss. The best foliar spray for reducing weight loss was the high level of irradiated K silicate which improved the weight loss by approximately 46.33% compared with that of the control. Additionally, the decay percentage increased with time during storage with maximum decay recorded at the end of the experiment. The differences in decay% among the successive weeks were 1.2%, 1.6%, 2.3% and 3.2% in the 1^st^ season and 1.8%, 1.2%, 2.3% and 3.3% in the 2^nd^ season. The highest percentage of decay was found between weeks three and four followed by weeks two and three. With respect to the foliar sprays used, the decay percentage followed the same pattern as the weight loss percentage where the highest decay percentage was obtained with ethrel, whereas the lowest decay percentage was achieved by spraying a high level of irradiated K silicate, which was approximately 50.91% less than that of the control group. There was no significant difference between the effects of K citrate and K silicate.

### Comparison between high and low concentration levels and irradiated and non-irradiated forms

High and low levels of either K citrate or K silicate had comparable effects on post-harvest weight loss and decay. Furthermore, both the irradiated and non-irradiated forms of K had comparable impacts on post-harvest decay percentage, even though the irradiated form was better than the non-irradiated form at reducing post-harvest weight loss.

D) **Effects of ethrel and potassium sprays on post-harvest berry firmness loss and shattering during storage:**

The data in Table 3 illustrate the effects of spraying with ethrel or different potassium sources on post-harvest changes in berry firmness and berry shattering percentage of Ruby Seedless grapevines. The berry firmness decreased significantly over time during storage, whereas berry shattering percentage increased. The deterioration in firmness was greater in the first two weeks of storage (3.6% and 3.2%, respectively) than in the 3^rd^ and 4^th^ weeks (2.8% and 3%, respectively). Compared with the control, all foliar sprays significantly reduced the deterioration of berry firmness and berry shattering throughout the four weeks of storage except for ethrel, where berry firmness loss and berry shattering significantly increased. The silicate form of the K spray significantly reduced the loss of firmness and shattering more than did the K citrate spray. The best results for berry firmness and shattering during the four weeks of storage were obtained with irradiated K silicate, particularly at high levels.

**Table 3:** Effects of spraying with ethrel or different potassium sources on post-harvest berry firmness (g/cm^2^) and berry shattering (%) in Ruby Seedless grapevines over a four-week storage period

	**2022**						**2023**					
	**Berry firmness(g/cm**^**2**^)											
**Weeks**	**0**	**1**	**2**	**3**	**4**	**Mean**	**0**	**1**	**2**	**3**	**4**	**Mean**
**Control**	41.60^p^	38.58^st^	35.93^v^	33.68^w^	31.22^x^	**36.20**^**I**^	43.89°	40.88^q^	38.09^s^	35.62^u^	33.19^v^	**38.33**^**I**^
**Ethrel**	39.34^rs^	36.49^uv^	33.73^w^	31.27^x^	29.51^y^	**34.07**^**J**^	40.85^q^	38.04^s^	35.62^u^	33.21^v^	30.89^w^	**35.72**^**J**^
**KC1**	45.72^lm^	42.38^p^	39.49^r^	37.02^u^	34.31^w^	**39.78**^**H**^	48.30^jk^	44.94^n^	41.83^p^	39.29^r^	36.59^t^	**42.19**^**H**^
**KC2**	47.44^jk^	44.08^no^	41.59^p^	38.40 ^t^	35.89^v^	**41.48**^**G**^	51.12^g^	47.59^k^	44.39^no^	41.58^p^	38.69^rs^	**44.67**^**G**^
**γKC1**	54.20^d^	50.25^h^	46.81^k^	43.81°	40.69^q^	**47.15**^**D**^	55.19^d^	51.20^g^	47.62^k^	44.88^n^	41.62^p^	**48.10**^**D**^
**γKC2**	55.67^c^	51.60^f^	48.11^j^	45.10^lm^	41.61^p^	**48.42**^**C**^	55.54^d^	51.70^g^	48.39^j^	45.04^n^	42.01^p^	**48.54**^**C**^
**KSi1**	51.12^fg^	47.39^jk^	44.17^no^	41.58^p^	38.39 ^t^	**44.53**^**F**^	51.74^g^	48.10^jk^	44.89^n^	42.05^p^	39.18 ^r^	**45.19**^**F**^
**KSi2**	53.09^e^	49.23^i^	45.88^l^	43.45°	39.41^rs^	**46.21**^**E**^	53.71^e^	50.03^h^	46.59^l^	44.89^n^	40.80^q^	**47.20**^**E**^
**γKSi1**	58.50^b^	54.22^d^	50.52^gh^	47.36^jk^	43.83°	**50.89**^**B**^	59.11^b^	55.02^d^	51.77^g^	48.05^jk^	43.81°	**51.59**^**B**^
**γKSi2**	59.36^a^	55.63^c^	51.02^f-h^	48.02^j^	44.91^mn^	**51.79**^**A**^	60.59^a^	56.41^c^	52.57^f^	49.29^i^	45.88^m^	**52.95**^**A**^
**Mean**	**50.60**^**A**^	**46.99**^**B**^	**43.73**^**C**^	**40.97**^**D**^	**37.98**^**E**^		**52.00**^**A**^	**48.41**^**B**^	**45.18**^**C**^	**42.39**^**D**^	**39.27**^**E**^	
	**Berry shattering (%)**											
**Weeks**	**0**	**1**	**2**	**3**	**4**	**Mean**	**0**	**1**	**2**	**3**	**4**	**Mean**
**Control**	0.00^w^	1.94^n^	2.11^m^	2.37^l^	5.25^e^	**2.33**^**B**^	0.00^w^	2.02°	2.99^k^	3.69^j^	6.79^e^	**3.10**^**B**^
**Ethrel**	0.00^w^	5.13^f^	11.85^c^	23.42^b^	29.12^a^	**13.90**^**A**^	0.00^w^	7.49^d^	14.66^c^	24.55^b^	34.03^a^	**16.15**^**A**^
**KC1**	0.00^w^	0.67^u^	1.39^p^	2.42^l^	5.48^d^	**1.99**^**C**^	0.00^w^	0.33^v^	1.42^qr^	2.46^lm^	5.51^f^	**1.94**^**C**^
**KC2**	0.00^w^	0.98^rs^	1.38^p^	2.13^m^	4.62^h^	**1.82**^**D**^	0.00^w^	0.33^v^	1.32^q-s^	2.29^mn^	5.16^g^	**1.82**^**DE**^
**γKC1**	0.00^w^	0.00^w^	1.08^r^	1.87^n^	4.26^i^	**1.44**^**F**^	0.00^w^	0.00^w^	1.14^st^	1.98°	4.42^i^	**1.51**^**F**^
**γKC2**	0.00^w^	0.00^w^	0.92^st^	1.60°	3.63^j^	**1.23**^**G**^	0.00^w^	0.00^w^	0.98^t^	1.69^p^	3.79^j^	**1.29**^**G**^
**KSi1**	0.00^w^	0.33^v^	1.26^q^	2.19^m^	4.97^g^	**1.75**^**E**^	0.00^w^	0.33^v^	1.34^q-s^	2.33^mn^	5.24^g^	**1.85**^**CD**^
**KSi2**	0.00^w^	0.67^u^	1.19^q^	2.09^m^	4.69^h^	**1.73**^**E**^	0.00^w^	0.33^v^	1.24^rs^	2.16^no^	4.85^h^	**1.72**^**E**^
**γKSi1**	0.00^w^	0.00^w^	0.87^t^	1.51°	3.42^k^	**1.16**^**H**^	0.00^w^	0.00^w^	0.92^t^	1.57^pq^	3.56^j^	**1.21**^**G**^
**γKSi2**	0.00^w^	0.00^w^	0.33^v^	0.57^u^	1.29^pq^	**0.44**^**I**^	0.00^w^	0.00^w^	0.67^u^	1.17^r-t^	2.61^l^	**0.89**^**H**^
**Mean**	**0.00**^**E**^	**0.97**^**D**^	**2.24**^**C**^	**4.02**^**B**^	**6.67**^**A**^		**0.00**^**E**^	**1.08**^**D**^	**2.67**^**C**^	**4.39**^**B**^	**7.60**^**A**^	

Different small letters indicate significant differences between interactions at *p* ≤ 0.05; different capital letters in rows and columns indicate significant differences between groups and storage periods at *p* ≤ 0.05; Duncan’s multiple range test. KC1: potassium citrate at 2000 ppm; KC2: potassium citrate at 4000 ppm; γKC1: irradiated potassium citrate at 750 ppm; γKC2: irradiated potassium citrate at 1500 ppm; KSi1: potassium silicate at 2000 ppm; KSi2: potassium silicate at 4000 ppm; γKSi1: irradiated potassium silicate at 750 ppm; γKSi1: irradiated potassium silicate at 1500 ppm.

### Comparison between high and low concentration levels and irradiated and non-irradiated forms

High-level sprays of K silicate and K citrate outperformed low level sprays in reducing the firmness loss and percentage of shattering, with the irradiated form performing better than the non-irradiated form.

E) **Effects of ethrel and potassium sprays on post-harvest TSS and acidity during storage:**

The data presented in Table 4, demonstrate the effects of ethrel and different potassium sprays on post-harvest changes in the TSS % and acidity% of Ruby Seedless grapevines. The data revealed that the TSS percentage and the rate of increase increased with time over the storage period. The average increases in the TSS values between each week and the previous week were 0.59%, 0.68%, 0.85%, and 0.92%, respectively. The TSS% was significantly increased by all of the foliar sprays used in comparison to the control, with ethrel obtaining the highest value. The acidity significantly decreased over the storage period for all foliar sprays used. Foliar K and ethrel sprays significantly reduced the acidity% compared with the control, with the lowest value obtained by ethrel. The highest acidity% was observed in the control treatment at harvest (0.590%), whereas the lowest value was obtained with ethrel foliar spray at the end of the storage period (0.343%). Moreover, silicate-based K sprays are more effective at increasing the TSS% and reducing the acidity% than K citrate sprays are. The best results for TSS% and acidity% were obtained with ethrel, followed by irradiated K silicate, particularly at high levels.

**Table 4:** Effects of spraying with ethrel or different potassium sources on post-harvest TSS (%) and acidity (%) of Ruby Seedless grapevines over a four-week storage period

	**2022**						**2023**					
	**TSS (%)**											
**Weeks**	**0**	**1**	**2**	**3**	**4**	**Mean**	**0**	**1**	**2**	**3**	**4**	**Mean**
**Control**	16.33^x^	16.85^vw^	17.49^rs^	18.35^lm^	19.25^g-i^	**17.66**^**G**^	16.10^x^	16.70^uv^	17.22^r^	18.12^lm^	19.01^h^	**17.43**^**I**^
**Ethrel**	17.93^no^	18.51^kl^	19.20^hi^	20.15^cd^	21.14^a^	**19.38**^**A**^	17.61^op^	18.20^l^	18.89^hi^	19.82^de^	20.79^a^	**19.06**^**A**^
**KC1**	16.70^w^	17.27^st^	17.93^no^	18.81^j^	19.71^f^	**18.09**^**F**^	16.31^wx^	16.87^tu^	17.50^p^	18.45^k^	19.25^fg^	**17.68**^**H**^
**KC2**	16.80^w^	17.37^s^	18.04^no^	18.91^j^	19.84^ef^	**18.19**^**E**^	16.51^vw^	17.04^r-t^	17.60^op^	18.57^jk^	19.38^f^	**17.82**^**G**^
**γKC1**	16.89^u-w^	17.47^rs^	18.12^mn^	19.04^ij^	19.95^de^	**18.29**^**D**^	16.61^v^	17.12^rs^	17.81^no^	18.68^ij^	19.60^e^	**17.96**^**F**^
**γKC2**	17.11^tu^	17.68^p-r^	18.35^lm^	19.25^g-i^	20.19^c^	**18.51**^**C**^	16.90^s-u^	17.48^pq^	18.14^lm^	19.03^gh^	19.96^cd^	**18.30**^**D**^
**KSi1**	16.80^w^	17.37^s^	18.03^no^	18.90^j^	19.81^ef^	**18.18**^**E**^	16.71^uv^	17.26^qr^	17.92^mn^	18.80^hi^	19.72 ^e^	**18.08**^**E**^
**KSi2**	17.03^uv^	17.63^qr^	18.27^m^	19.18^hi^	20.11^cd^	**18.44**^**C**^	16.90^s-u^	17.47^pq^	18.14^lm^	19.03^gh^	19.98^cd^	**18.30**^**D**^
**γKSi1**	17.30 ^st^	17.81^o-q^	18.50^kl^	19.38^gh^	20.33^bc^	**18.66**^**B**^	17.12^rs^	17.62^op^	18.35^kl^	19.25^fg^	20.17^bc^	**18.50**^**C**^
**γKSi2**	17.34^s^	17.89^n-p^	18.58^k^	19.46^g^	20.44^b^	**18.74**^**B**^	17.20^r^	17.79^no^	18.46^jk^	19.36^f^	20.31^b^	**18.63**^**B**^
**Mean**	**17.02**^**E**^	**17.59**^**D**^	**18.25**^**C**^	**19.14**^**B**^	**20.08**^**A**^		**16.80**^**E**^	**17.35**^**D**^	**18.00**^**C**^	**18.91**^**B**^	**19.82**^**A**^	
	**Acidity (%)**											
**Weeks**	**0**	**1**	**2**	**3**	**4**	**Mean**	**0**	**1**	**2**	**3**	**4**	**Mean**
**Control**	0.590^a^	0.551^de^	0.524^fg^	0.488^jk^	0.469^lm^	**0.525**^**A**^	0.570^a^	0.532^de^	0.513^fg^	0.457^l-n^	0.439^n-q^	**0.502**^**A**^
**Ethrel**	0.441^op^	0.419^r^	0.392^s^	0.368^t^	0.343^u^	**0.393**^**I**^	0.431^p-r^	0.398^u^	0.380^v^	0.355^x^	0.333^y^	**0.380**^**H**^
**KC1**	0.582^ab^	0.545^de^	0.519^fg^	0.473^kl^	0.453^m-p^	**0.514**^**B**^	0.562^ab^	0.513^fg^	0.501^gh^	0.455^l-n^	0.437^o-r^	**0.494**^**B**^
**KC2**	0.569^bc^	0.536^ef^	0.518^fg^	0.461^l-n^	0.442^n-p^	**0.505**^**C**^	0.550^bc^	0.509^fg^	0.487^h-j^	0.446^m-p^	0.428^p-r^	**0.484**^**C**^
**γKC1**	0.510^gh^	0.472^kl^	0.454^l-p^	0.421^qr^	0.392^s^	**0.450**^**F**^	0.531^de^	0.490^hi^	0.470^j-l^	0.431^p-r^	0.418^r-t^	**0.468**^**E**^
**γKC2**	0.508^g-i^	0.466^lm^	0.444^n-p^	0.402^s^	0.390^s^	**0.442**^**G**^	0.521^ef^	0.479^ij^	0.454^l-o^	0.423^qr^	0.400^u^	**0.456**^**F**^
**KSi1**	0.561^cd^	0.522^fg^	0.500^h-j^	0.458^l-o^	0.438^pq^	**0.496**^**D**^	0.552^bc^	0.511^fg^	0.485^h-j^	0.446^m-p^	0.427^qr^	**0.484**^**C**^
**KSi2**	0.551^de^	0.510^gh^	0.492^ij^	0.453^m-p^	0.436^p-r^	**0.488**^**E**^	0.541^cd^	0.501^gh^	0.476^i-k^	0.439^n-q^	0.420^q-s^	**0.475**^**D**^
**γKSi1**	0.491^j^	0.453^n-p^	0.442^n-p^	0.400^s^	0.389^s^	**0.435**^**G**^	0.461^k-m^	0.425^qr^	0.404^s-u^	0.374^vw^	0.358 ^wx^	**0.404**^**G**^
**γKSi2**	0.471^k-m^	0.440^op^	0.422^qr^	0.388^s^	0.370^t^	**0.418**^**H**^	0.460^k-m^	0.419^r-t^	0.402^tu^	0.370^v-x^	0.356^x^	**0.402**^**G**^
**Mean**	**0.527**^**A**^	**0.491**^**B**^	**0.471**^**C**^	**0.431**^**D**^	**0.412**^**E**^		**0.518**^**A**^	**0.478**^**B**^	**0.457**^**C**^	**0.420**^**D**^	**0.402**^**E**^	

Different small letters indicate significant differences between interactions at P ≤ 0.05; different capital letters in rows and columns indicate significant differences between groups and storage periods at P ≤ 0.05; Duncan’s multiple range test. KC1: potassium citrate at 2000 ppm; KC2: potassium citrate at 4000 ppm; γKC1: irradiated potassium citrate at 750 ppm; γKC2: irradiated potassium citrate at 1500 ppm; KSi1: potassium silicate at 2000 ppm; KSi2: potassium silicate at 4000 ppm; γKSi1: irradiated potassium silicate at 750 ppm; γKSi1: irradiated potassium silicate at 1500 ppm

### Comparison between high and low concentration levels and irradiated and non-irradiated forms

High levels of both K sources outperformed low levels in increasing the TSS percentage and reducing the acidity percentage throughout the four weeks of storage. Additionally, the irradiated form of the K source has a more positive effect on the TSS% and acidity% than non-irradiated K source does.

F) Effects of ethrel and potassium sprays on post-harvest total sugars and total anthocyanin contents during storage:

The foliar sprays under investigation had a substantial effect on the total sugars% and anthocyanin contents of the berries throughout the storage period, and the values increased dramatically as the storage time increased (Table 5). Moreover, the rate of increase in total sugars percentage increased from one week to the next with values of 3.3% and 3.8% for the first two weeks and 4.9% for the last two weeks. Additionally, the rates of increase in anthocyanin content throughout the four weeks of storage were 3.4%, 3.9%, 4.9% and 4.9%, respectively, which were similar to the rates of increase in total sugar%. All foliar sprays significantly increased the total sugars and anthocyanin contents compared with the control. The foliar spray of ethrel resulted in the highest total sugars percentage and anthocyanin content, while the lowest values were obtained in the control. For K sprays, the silicate form was superior to the citrate form in increasing the total sugars and anthocyanin contents. The best K spray after ethrel was irradiated K silicate, particularly at high levels.

**Table 5:** Effects of spraying with ethrel or different potassium sources on post-harvest total sugars (%) and total anthocyanin (mg/100 g FW) in Ruby Seedless grapevines over a four-week storage period

	**2022**						**2023**					
	**Total sugars (%)**											
**Weeks**	**0**	**1**	**2**	**3**	**4**	**Mean**	**0**	**1**	**2**	**3**	**4**	**Mean**
**Control**	11.88^z^	12.27^y^	12.73^x^	13.37^w^	14.03^uv^	**12.86**^**G**^	12.43^z^	12.86^y^	13.34^x^	13.97^w^	14.69^st^	**13.46**^**I**^
**Ethrel**	15.84^i-k^	16.33^f-h^	16.97^e^	17.80^b^	18.60^a^	**17.11**^**A**^	15.95^m^	16.51^j-l^	17.17^ef^	17.95^bc^	18.83^a^	**17.28**^**A**^
**KC1**	13.83^v^	14.29^tu^	14.87^qr^	15.53^k-m^	16.33^f-h^	**14.97**^**F**^	14.12^vw^	14.56^t^	15.15^pq^	15.93^m^	16.63^i-k^	**15.28**^**H**^
**KC2**	13.92^v^	14.37^st^	14.90^qr^	15.63^j-l^	16.40^fg^	**15.04**^**F**^	14.27^uv^	14.73^r-t^	15.31^op^	16.03^m^	16.87^g-i^	**15.44**^**G**^
**γKC1**	14.66^rs^	15.20^n-p^	15.73^j-l^	16.50^fg^	17.33^cd^	**15.88**^**D**^	14.93^q-s^	15.43^op^	16.01^m^	16.77^h-j^	17.63^d^	**16.16**^**D**^
**γKC2**	14.78^r^	15.27^m-o^	15.87^ij^	16.57^f^	17.40^c^	**15.98**^**D**^	14.97^q-s^	15.43^op^	16.03^m^	16.90^f-i^	17.71^cd^	**16.21**^**D**^
**KSi1**	14.39^st^	14.89^qr^	15.47^l-n^	16.23^gh^	17.00^e^	**15.60**^**E**^	14.52^tu^	14.99^qr^	15.57^no^	16.31^l^	17.10^e-g^	**15.70**^**F**^
**KSi2**	14.48^st^	14.96^p-r^	15.53^k-m^	16.33^f-h^	17.10^de^	**15.68**^**E**^	14.69^st^	15.15^pq^	15.73^mn^	16.53^j-l^	17.33^e^	**15.89**^**E**^
**γKSi1**	15.12^o-q^	15.63^j-l^	16.20^gh^	16.97^e^	17.73^b^	**16.33**^**C**^	15.42^op^	15.93^m^	16.53^j-l^	17.32^e^	18.19^b^	**16.68**^**C**^
**γKSi2**	15.54^k-m^	16.07^hi^	16.63^f^	17.44^c^	18.33^a^	**16.80**^**B**^	15.84^mn^	16.37^kl^	16.96^f-h^	17.83^cd^	18.70^a^	**17.14**^**B**^
**Mean**	**14.44**^**E**^	**14.93**^**D**^	**15.49**^**C**^	**16.24**^**B**^	**17.03**^**A**^		**14.71**^**E**^	**15.20**^**D**^	**15.78**^**C**^	**16.55**^**B**^	**17.37**^**A**^	
	**Total anthocyanin (mg/100 g FW)**											
**Weeks**	**0**	**1**	**2**	**3**	**4**	**Mean**	**0**	**1**	**2**	**3**	**4**	**Mean**
**Control**	27.30^z^	28.23^y^	29.14^x^	30.77^v^	32.16^t^	**29.52**^**I**^	28.09^z^	29.07^y^	30.16^x^	32.23^w^	33.19^v^	**30.55**^**J**^
**Ethrel**	39.22^i^	40.51^g^	42.15^d^	44.21^b^	46.39^a^	**42.50**^**A**^	40.32^ij^	41.69^g^	43.27^e^	45.39^c^	47.61^a^	**43.66**^**A**^
**KC1**	29.11^x^	30.10^w^	31.28^u^	32.83^s^	34.41^q^	**31.55**^**H**^	32.26^w^	33.36^v^	34.62^tu^	36.03^qr^	38.46^m-o^	**34.95**^**I**^
**KC2**	31.21^u^	32.78^s^	33.53^r^	35.29^p^	36.90^n^	**33.94**^**G**^	33.14^v^	34.27^u^	35.53^rs^	37.31^p^	39.14^k-n^	**35.88**^**H**^
**γKC1**	34.37^q^	35.33^p^	36.90^n^	38.73^jk^	40.62^fg^	**37.19**^**E**^	36.24^qr^	37.48^p^	38.90^l-n^	40.80^hi^	42.79^ef^	**39.24**^**E**^
**γKC2**	35.22^p^	36.41°	37.90^l^	39.71^h^	41.63^e^	**38.17**^**D**^	37.88^op^	39.21^k-m^	40.62^hi^	42.64^ef^	44.71^cd^	**41.01**^**D**^
**KSi1**	32.13^t^	33.09^s^	34.25^q^	36.37°	37.87^l^	**34.74**^**F**^	35.25^st^	36.43^q^	37.86^op^	39.65^j-l^	41.61^g^	**38.16**^**G**^
**KSi2**	34.22^q^	35.58^p^	36.82^n^	38.57^k^	40.49^g^	**37.14**^**E**^	35.97^q-s^	37.46^p^	38.42^no^	40.51^hi^	42.49^f^	**38.97**^**F**^
**γKSi1**	36.33°	37.55^lm^	39.03^ij^	40.97^f^	42.97^c^	**39.37**^**C**^	38.44^no^	39.75^jk^	41.25^gh^	43.26^e^	45.35^c^	**41.61**^**C**^
**γKSi2**	37.34^m^	38.93^i-k^	40.55^g^	42.07^d^	44.17^b^	**40.62**^**B**^	39.20^k-m^	40.57^hi^	42.47^f^	44.14^d^	46.31^b^	**42.54**^**B**^
**Mean**	**33.65**^**E**^	**34.85**^**D**^	**36.16**^**C**^	**37.95**^**B**^	**39.76**^**A**^		**35.68**^**E**^	**36.93**^**D**^	**38.31**^**C**^	**40.20**^**B**^	**42.17**^**A**^	

Different small letters indicate significant differences between interactions at *p* ≤ 0.05; different capital letters in rows and columns indicate significant differences between groups and storage periods at *p* ≤ 0.05; Duncan’s multiple range test. KC1: potassium citrate at 2000 ppm; KC2: potassium citrate at 4000 ppm; γKC1: irradiated potassium citrate at 750 ppm; γKC2: irradiated potassium citrate at 1500 ppm; KSi1: potassium silicate at 2000 ppm; KSi2: potassium silicate at 4000 ppm; γKSi1: irradiated potassium silicate at 750 ppm; γKSi1: irradiated potassium silicate at 1500 ppm

### Comparison between high and low concentration levels and irradiated and non-irradiated forms

The effect of higher levels of K sources on total sugars% is similar to the effect of the lower level in 2022, whereas in 2023, its effect surpassed the lower level. In terms of the anthocyanin content, high-K sprays induced a greater increase than did low-K sprays. The irradiated K sprays outperformed the non-irradiated sprays in increasing the total sugars and anthocyanin contents during the storage period.

### Overall performance of the potassium treatments vs. the control and ethrel

Foliar K sprays surpassed the control and ethrel in increasing cluster weight and berry weight and firmness and reducing weight loss, decay, firmness loss and shattering during the four-week storage period. On the other hand, foliar K sprays increased the TSS, total sugars and total anthocyanin contents and decreased acidity compared with the control; however, ethrel was more effective.

## Discussion

Our data revealed that, compared with the control, all the potassium forms and ethrel foliar sprays increased cluster and berry weights and berry firmness, with K sprays being superior to ethrel. The positive effects of K silicate treatments on the cluster and berry weights and berry firmness of Ruby Seedless grapes were also confirmed in Thompson seedless grapevines [27] and ARRA15 table grapes [28], where foliar spraying with potassium silicate considerably increased the cluster weight, average berry weight and berry hardness in comparison with those of the control. Additionally, foliar application of K citrate to Thompson seedless grape increased cluster and berry weights compared to the control [29]. Similarly, applying ethrel topically to the leaves of Flame Seedless grapevines greatly improved the cluster and berry weight [30]. The involvement of K in improved photosynthesis and cell division may be the reason for the increase in berry weight in response to various potassium sprays [31]. The reduced firmness in response to ethrel was linked to the faster breakdown of pectin and the inhibition of cellulose synthesis [32]. The interaction between silicon and potassium within the plant, which stabilizes cell membranes, encourages cell elongation, and increases resilience to stress conditions, is likely the reason why the silicate form of K has a greater effect than the citrate form [33].

The positive effects of ethrel and potassium foliar sprays on total soluble solids, total sugars, total anthocyanin and acidity were also observed in ARRA15 grapevine [28], and Red Roomy grapevine [34], where spraying potassium silicate increased the TSS%, total sugar, and anthocyanin contents and decreased acidity%, whereas spraying potassium schoenite increased the TSS% and anthocyanin contents and decreased the acidity% in Crimson Seedless grapevine [35]. Similarly, ethrel foliar spray was found to lower the acidity percentage of Flame Seedless grapevine [30, 36] and Crimson Seedless grapevine [35] while dramatically increasing the TSS%, anthocyanin, and vitamin C contents. Additionally, Hegazi et al. [37], reported that spraying Flame Seedless grapes with ethephon resulted in anthocyanin content than did the control. Moreover, the content of anthocyanin increased as the level of ethephon increased. The increase in the TSS and decrease in acidity during storage were also confirmed by Mirdehghan and Rahimi [38], Mohamed et al. [39], and Sortino et al. [40], who reported that the TSS% increased gradually and significantly, whereas the total acidity decreased gradually and significantly with prolonged storage. The ability of potassium and silicon to increase the TSS percentage and decreasing the acidity% during storage over the control was also confirmed by Elmehrat et al. [28], in ARRA 15 grapes, where potassium silicate-treated vines presented a greater percentage of berry sugars than did the control at the beginning of cold storage and at the end of the storage period. The reduction of tartaric acid upon conversion to potassium tartarate may be the reason for the function of potassium in lowering acidity levels in berries [41, 42]. Potassium has multiple functions that play a role in increasing sugar and anthocyanin contents in fruits. First, potassium up-regulates genes responsible for sugar metabolism and anthocyanin synthesis. It also positively influences sugar synthesis, actively contributing to its transport from the leaves to the fruits, which are then used as a substrate for anthocyanin production. Furthermore, potassium activates enzymes involved in anthocyanin synthesis pathways [43, 44]. Ethrel outperformed all K foliar sprays in increasing TSS, sugars, and anthocyanin contents while reducing acidity. This effect is attributed to increased ethylene production, which stimulates the onset of grape ripening and, at a greater rate, triggers a series of physiological changes that lead to increased sweetness. Additionally, ethrel increased the expression of genes involved in sugar transport and sucrose cleavage resulting in accelerated sugar accumulation in the berries. The increased anthocyanin content could be attributed to ethrel activation of different genes and proteins involved in anthocyanin biosynthesis and transport such as *VACUOLAR INVERTASE* (*vINV*) and *VvERF75* [45] and [32]) which promote fruit ripening by promoting ethylene biosynthesis and chlorophyll degradation [46].

Our results revealed reduced weight loss and decay in response to different K sprays. These results were consistent with those of Elmehrat et al. [28], on ARRA15 grapes and Abdelaziz et al. [47] on ARRA18, as they reported that spraying with potassium silicate decreased weight loss and decay. Moreover, Zhang et al. [48], found that, potassium silicate greatly reduced the percentage of weight loss and decay incidence of table grapes during cold storage when compared to the control. Additionally, the percentage of decay and weight loss was reduced when Crimson Seedless grapevines were sprayed with potassium schoenite or ethrel [35]. These findings could be attributed to the role of potassium in stomatal closure, which reduces fruit transpiration and respiration rates, and may explain the reduced weight loss [49]. Moreover, silicon absorbed by plants is deposited on epidermal tissues, resulting in an increase in plant tissue rigidity [50] and increasing the resistance of plant cell walls to degradation [51].

A reduction in berry firmness during storage was also reported by Mirdehghan and Rahimi [38] for the Rishbaba’ and ‘Olhoghi’ cultivars; Mohamed et al. [39], for mango and Sortino et al. [40], for Red Globe, as they found that the berry firmness of table grapes decreased gradually and significantly with the prolonged cold storage to reach its minimum value at the end of storage. The superior effect of different potassium sources over the control and ethrel in reducing berry softening was also reported by Elmehrat et al. [28], they reported that spraying ARRA15 vines with potassium silicate significantly reduced the berry softening rate compared with that of the control. Additionally, Zhang et al. [48], demonstrated that preharvest silicon fertilization significantly increased Red Globe and Black Monukka table berry firmness at harvest. Moreover, Takma and Korel [52] reported that pre-harvest treatment with alginate prevents deterioration of grape berry firmness during cold storage.

The remarkable results obtained with the use of irradiated potassium compounds can be attributed to the increased rate of their degradation, which results in a form that is readily available to the plant. It has been proven that the use of radiation leads to an increased rate of material degradation. This may be explained by the high energy that gamma radiation delivers, which is greater than the energy required to break down any organic bond and has the capacity to penetrate a material’s full thickness [53]. Radiation, particularly gamma rays, can be used to quickly and efficiently breakdown complex materials such as salts and improve their availability. Compost that had been exposed to radiation had better degradation than non-irradiated compost, which made the elements more accessible and easier for plants to absorb [16]. There was also more dissolved organic matter and better organic N mineralization because of the alternation of organic N forms, the breakdown of complex stable organic-N compounds, and greater availability of N and P [54]. Additionally, the beneficial effects of sludge are amplified when radiation exposure increases [55], resulting in the release of NH4+ and an increase in N bioavailability [56]. Numerous compounds have demonstrated impressive effectiveness in improving plant characteristics and fruit quality when exposed to radiation. The use radiation-degraded alginate improves Zea maize plant quality and production [57]. When irradiated kappa carrageenan was applied to peanuts, the number and yield of pods and seeds increased, leading to increased productivity [58]. Additionally, irradiated kappa and iota carrageenan solutions were used to accelerate the growth of rice seedlings grown hydroponically [59]. Additionally, irradiated K-carrageenan significantly increased the marketable and total weight of pechay plants over non-irradiated carrageenan which was attributed to the higher absorption rate, as irradiation decreased the particle size, increasing the amount of K-carrageenan absorbed by plants [20]. Furthermore, the addition of irradiated chitosan at 75–100 kGy accelerated the growth of strawberries, lisianthus, limonium, and chrysanthemum. This improvement may be explained by the fact that when radiation exposure increased, the molecular mass of chitosan in solution decreased [60].

Gamma radiation accelerates the degradation of potassium citrate, as evidenced by multiple resonance signals in an electron spin resonance investigation [15]; potassium compound crystals, as demonstrated by a slight peak shift indicating the formation of defect centers [61]; potassium chlorate [62]; and potassium iodate, where the activation energy decreases upon irradiation and the decomposition rate increases with the irradiation dose. He attributed the increased rate of thermal disintegration under irradiation to the combined impacts of enlarged lattice defects, displacements, and chemical degradation [63].

## Conclusions

All potassium sprays improved the physical traits of Ruby Seedless grapes at harvest and during storage—berry weight and firmness increased at harvest, while berry weight loss, decay, shattering, and firmness loss decreased during storage—when compared to the control and ethrel. Although ethrel proved more effective, potassium sprays also enhanced the berries’ chemical characteristics by increasing their sugar, anthocyanin, and total soluble solids content while decreasing their acidity at harvest and during storage compared to the control. In terms of improving the physical and chemical characteristics of seedless Ruby grapes, high potassium levels outperformed low potassium levels, potassium silicate outperformed potassium citrate, and gamma-irradiated potassium sprays outperformed non-irradiated potassium sprays. Gamma-irradiated potassium silicate may be a suitable alternative to ethrel at a concentration of 1500 ppm, ensuring fruit of superior quality and longer shelf life.

## Data Availability

The data that support the findings of this study are not openly available due to reasons of sensitivity and are available from the corresponding author upon reasonable request
